# Unraveling the Puzzle: Health Benefits of Probiotics—A Comprehensive Review

**DOI:** 10.3390/jcm13051436

**Published:** 2024-03-01

**Authors:** Sabiha Gul, Emanuele Durante-Mangoni

**Affiliations:** 1 Department of Precision Medicine, University of Campania “Luigi Vanvitelli”, Via de Crecchio 7, 80138 Napoli, Italy; sabiha.gul@unicamapnia.it; 2 Unit of Infectious & Transplant Medicine, A.O.R.N. Ospedali dei Colli—Ospedale Monaldi, Piazzale Ettore Ruggieri, 80131 Napoli, Italy

**Keywords:** probiotics, health benefits, common microbes, mechanism of action

## Abstract

A growing number of probiotic-containing products are on the market, and their use is increasing. Probiotics are thought to support the health of the gut microbiota, which in turn might prevent or delay the onset of gastrointestinal tract disorders. Obesity, type 2 diabetes, autism, osteoporosis, and some immunological illnesses are among the conditions that have been shown to possibly benefit from probiotics. In addition to their ability to favorably affect diseases, probiotics represent a defense system enhancing intestinal, nutritional, and oral health. Depending on the type of microbial strain utilized, probiotics can have variable beneficial properties. Although many microbial species are available, the most widely employed ones are lactic acid bacteria and bifidobacteria. The usefulness of these bacteria is dependent on both their origin and their capacity to promote health. Probiotics represent a valuable clinical tool supporting gastrointestinal health, immune system function, and metabolic balance. When used appropriately, probiotics may provide benefits such as a reduced risk of gastrointestinal disorders, enhanced immunity, and improved metabolic health. Most popular probiotics, their health advantages, and their mode of action are the topic of this narrative review article, aimed to provide the reader with a comprehensive reappraisal of this topic matter.

## 1. Introduction

Since the human microbiota plays a well-established role in both health and disease, it is becoming more and more important to develop strategies to shape a healthier human microbiota [1]. Following the discovery of the correlation between gut microbiota and health, numerous studies were carried out to determine the exact mechanism underlying this association. It has become clear that individual bacterial species within the gut microbiota tend to be positively correlated with an overall better health status [2].

Probiotics have gained significant interest over the past few decades because of their potential to improve health and even treat certain diseases when used in conjunction with other therapies [1]. To describe such beneficial effects of gut bacteria, the term ‘probiotics’ was introduced for the first time in 1965 by Stillwell and Lilley [3]. At the beginning of the 20th century, the properties of fermented dairy products had been noticed by the Nobel laureate Élie Metchnikoff in Paris. He postulated that fermented dairy products included lactic acid bacteria, which supported the health of the immune system and increased longevity [4].

Probiotics were initially thought to exert a favorable effect on gut microbiota composition, but subsequent studies have shown that they can also positively connect with many chronic health conditions [5]. Every bacterial strain has unique qualities; some may help in improving diabetes mellitus (DM), others in managing obesity, and some in treating osteoporosis. Apart from these widely recognized effects, numerous research studies have suggested the significance of probiotics in the contexts of autism, irritable bowel syndrome (IBS), and wound healing [6,7,8].

Because of their positive effects on gut homeostasis, probiotics are increasingly used as a food supplement. Uncovering the processes behind host-gut microbial interactions has been the focus of numerous efforts [9,10]. Although the effects and processing mechanisms of probiotics are not always evident, using them to reduce disease pathology is an active area of current scientific investigation. Furthermore, there are a few safety concerns that need to be addressed [11], specifically for immunocompromised individuals such as those with HIV/AIDS, those undergoing chemotherapy or receiving immunosuppressive medications following organ transplantation, preterm infants, particularly those with extremely low birth weights or other health complications, critically ill patients, elderly individuals with severe comorbidities, and older adults with significant underlying health conditions, especially those affecting immune function or gastrointestinal health. These populations are all highly prone to adverse events when taking probiotics [12]. These include the possibility that certain probiotic strains may transfer intrinsic virulence factors and/or antimicrobial drug resistance determinants. Additional concerns entail (infrequent) adverse reactions, such as metabolic disruptions, urinary tract infections, sepsis, opportunistic infections, and ischemia. The absence of thorough clinical trials and the deficiency of comprehensive and unambiguous clinical recommendations relating to probiotics are additional reasons for concern [13].

Probiotics should be differentiated from other related compounds, classified into the following categories.

### 1.1. Prebiotics

Prebiotics are non-digestible food ingredients that beneficially affect the host by selectively stimulating the growth and/or activity of one or a limited number of bacteria in the colon. The substrate must not be hydrolyzed or absorbed in the stomach or small intestine; it must be selective for beneficial commensal bacteria in the large intestine such as the bifidobacteria. Fermentation of the substrate should induce beneficial luminal/systemic effects within the host. The rationale behind prebiotic use is to elevate the endogenous numbers of beneficial bacterial strains including *Lactobacilli* and *Bifidobacteria*. This increase in beneficial microbes will impart the beneficial effects seen as a result of probiotic administration, including an increase in SCFA production, particularly butyrate, which can provide fuel for enterocytes, prevent pathogenic bacteria adherence, produce anti-bacterial substances, and decrease luminal pH. Examples of prebiotics are inulin and resistant starches (legumes, vegetables, and cereals), lactulose (lactose synthetic), germinated barley foodstuff, glyco-oligosaccharides, stachyose, and gentio-oligosaccharides [14].

### 1.2. Synbiotics

The International Scientific Association for Probiotics and Prebiotics has updated the concept of synbiotics according to a panel of experts. According to them, there are two types of synbiotics: complementary and synergistic. A complementary synbiotic consists of a probiotic and a prebiotic that together confer one or more health benefits, but do not require co-dependent functions. A synergistic synbiotic contains a substrate that is selectively utilized by co-administered microorganism(s) [14]. Synbiotics might be more active than either a probiotic or prebiotic alone in preventing GI disorders. The potential benefits of synbiotic therapy are obvious; however, the great challenge, as is the case with probiotics and prebiotics alone, is to determine the best combination for each disease setting and patient. The first attempts should involve combining probiotics and prebiotics which have demonstrated individual benefits to determine if there are additive effects; alternatively, a more structured approach would be to determine the specific properties that a prebiotic requires to be beneficial to the probiotic, and select the prebiotic accordingly. Examples of synbiotics include *Bifidobacteria + Fructo-oligosaccharides*, *Lactobacilli + Lactitol*, and *Bifidobacteria + Galacto-oligosaccharides* [15].

### 1.3. Designer Probiotics

Designer probiotics, also known as genetically modified probiotics, are microorganisms that have been genetically engineered to possess specific traits or functionalities beyond what is naturally found in probiotic strains. These pharmabiotics can be designed to address various health issues or provide additional benefits beyond conventional probiotics. One example of a designer probiotic is an engineered variant of *Lactococcus lactis* (LAB), a commonly used probiotic bacterium, to produce therapeutic proteins for the treatment of inflammatory bowel disease (IBD), diabetes, and type I allergies. In this example, *Lactococcus lactis* is genetically modified to express and deliver anti-inflammatory molecules directly to the inflamed intestinal tissue [16].

The goal of this narrative review is to present a thorough summary of the state of research on probiotics, highlighting the importance of having a sophisticated understanding of both their mechanisms of action and health benefits. We aim to add to the ongoing discussion on probiotics and their potential to improve human health by critically analyzing existing literature.

## 2. Most Common Probiotics and Their Selection

**Figure 1:**
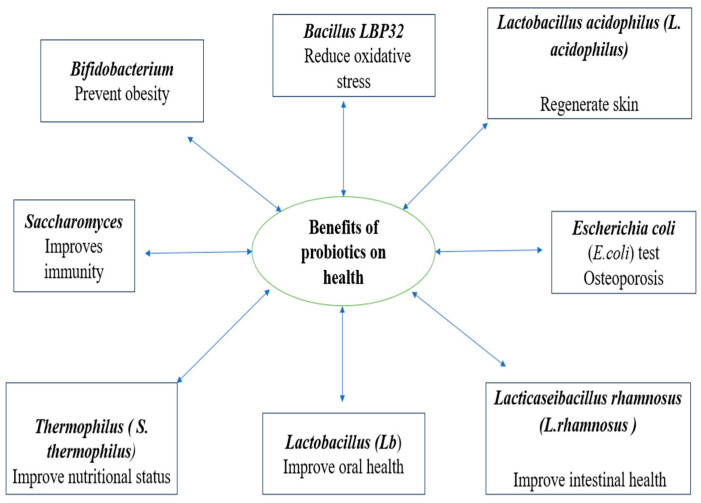
Highlights of the health advantages of several probiotics [1].

A growing body of research is being done on the use of probiotics, which are defined as “live microorganisms that, when administered in adequate amounts, confer a health benefit to the host” [17]. Probiotics can be utilized for a variety of illnesses depending on their genetic makeup, the number of species comprised in the product, its intended use, and its shelf life. This is because probiotics have diverse nutritional and therapeutic qualities. A probiotic strain’s production, effects, and health advantages for the host determine which strain to use (Figure 1) [1]. *Lactobacillus*, *Escherichia coli*, *Bifidobacterium*, *Enterococcus*, *Saccharomyces*, *Pediococcus*, *Lactobacillaceae*, *Streptococcus*, and *Leuconostoc* strains of probiotics have been utilized in an attempt to obtain defined health benefits. The two most popular microorganisms used as probiotics are *Bifidobacterium* spp. and *lactic acid bacteria* (LAB) [18].

For a food to be considered probiotic, it must contain 10^6^ CFU/g of probiotic microorganisms. 10^7^–10^9^ CFU should be taken daily for human consumption. Furthermore, it is well recognized that the strain of probiotic food a product contains determines how much of it should be consumed [19]. Typically, saliva samples are used to derive strains of *Lactobacillus* species, such as *Lacticaseibacillus paracasei* (*L. paracasei*), *Lactiplantibacillus paraplantarum* (*L. Plantarum*), and *Lacticaseibacillus rhamnosus* (*L. rhamnosus*). These anaerobes, which are also found in the oral cavity and breast milk, are known as bifidobacterial species. They are thought to be safe [16]. The strain must have originated from the target and natural microflora, since this will ensure its survival in the acidic environment of the stomach during transit [20]. Since curd is consumed all over the world, it is said to be the best source of probiotics [21]. Additionally, the biosafety level determines which strains are chosen; the chosen strains should not be hazardous or pathogenic [13]. The following safety characteristics need to be verified: hemolytic activity, antibiotic susceptibility, and antibiotic resistance gene carriage. The production of bacteriocins by probiotics is, in fact, an essential part of their defense against infections; yet, overuse of antibiotics may harm the gut microbiota in general [21]. The ability of various strains to withstand bile concentrations also varies. Higher quantities of bile may inhibit the development of the ingested strains [19]. By changing the pH of their surroundings, probiotics can outcompete any pathogen that may be present. Probiotics are similar to pathogens in that they attach to mucosal adhesion sites, reducing both the likelihood that pathogens will adhere and the likelihood that probiotics will be washed out [20].

Moreover, in the past, choosing a probiotic strain has mostly depended on its capacity to produce bacteriocin. Certain bacteria, including probiotic strains, produce antimicrobial peptides or proteins known as bacteriocins, which inhibit the growth of rival or closely related species. These probiotics are mentioned below, along with the names of the bacteriocins they are known to produce [22].

### 2.1. Lactobacillus acidophilus

*Lactacin F* bacteriocin is produced by some strains of *L. acidophilus* and exhibits antimicrobial activity against various pathogens, including other *Lactobacillus* species and certain Gram-positive bacteria [22,23].

### 2.2. Lactiplantibacillus plantarum

*L. plantarum* produces several different *plantaricins*, including *plantaricin S*, *plantaricin EF*, and *plantaricin W*, which have broad-spectrum antimicrobial activity against various Gram-positive and Gram-negative bacteria [22,23].

### 2.3. Lacticaseibacillus casei

Certain strains of *Lacticaseibacillus casei* (*L. casei*) produce *caseicins*, which are bacteriocins effective against other *Lactobacillus* species and some pathogenic bacteria such as *Escherichia coli*, *Staphylococcus aureus*, *Salmonella* spp., *Bacillus cereus*, and *Clostridium* spp. [22,23].

### 2.4. Ligilactobacillus salivarius

*L. salivarius* strains produce several *salivaricins*, including *salivaricin A*, *B*, and *F*, which have antimicrobial activity against oral pathogens such as *Streptococcus mutans* and *Streptococcus pyogenes* [22,23].

### 2.5. Limosilactobacillu reuteri

Although not a traditional bacteriocin, *reuterin* is a potent antimicrobial compound produced by *L*. *reuteri*. It is a broad-spectrum antimicrobial compound with activity against a wide range of bacteria, fungi, and protozoa [22,23].

### 2.6. Bifidobacterium *spp.*

Some strains of *Bifidobacterium* spp. produce bacteriocins known as *bifidocins*, which possess antimicrobial activity against bacteria including *E*. *coli*, *L*. *monocytogenes*, and *S*. *aureus*, as well as some yeasts and competing gut microbes [22,23].

### 2.7. Pediococcus pentosaceus

*Pediococcus pentosaceus* (*P. pentosaceus*), as a kind of *Lactococcus lactis* (*LAB*), has numerous probiotic effects. PE-ZYB1 is a new bacteriocin generated by *P*. *pentosaceus* zy-B, isolated from *Mimachlamys nobilis*, with antimicrobial activity against *L*. *monocytogenes*. This bacteriocin could be used in the seafood industry as an important weapon against marine animal-associated bacteria [22,23].

Probiotics have been shown to alter the intestinal microbiota found in the gut and to have a positive impact on the host’s immune system [24]. By altering the humoral and cellular immune responses, probiotics are thought to strengthen the immune system [20]. Various methods, such as molecular and biochemical tests, are employed to identify the bacterial strain genus. Subsequently, procedures such as PCR and gene sequencing tests are employed to distinguish between strains of the same species [21,22,23,24,25]. Further tests are carried out, such as platelet aggregation tests, which are vital indicators of pathogen activity, and hemolytic tests, which establish if the organism is able to destroy red blood cells [26].

Probiotic food products should preferably be stored at a temperature of 4–5 °C. The product should be used according to the label’s instructions, which should not contain any deceptive information [27].

## 3. Major Health Effects of Probiotics

### 3.1. On Nutritional Status

People’s concerns regarding their health are very significant nowadays. Around a century ago, probiotic use and its potential health advantages were first explored, aiming to prevent disease. Fermented foods and beverages have a long and important cultural and culinary history. Now, fermented foods have gained recognition for their nutritional value after the addition of probiotics. These meals are typically prepared from nutrient-dense base ingredients including milk, meat, grains, and legumes, which are naturally high in protein, vitamins, and minerals. Particularly, emerging research indicates that the live microorganisms included in fermented meals support the health of the digestive system and the body as a whole [28].

It is now a common practice to add specific probiotic microorganisms to fermented foods. Thus, many of the commercial yogurt and cultured milk products now contain probiotic strains of *Lactobacilli* and *Bifidobacteria*. In these applications, the fermented food becomes the delivery vehicle for the probiotic. Moreover, most cheeses and yogurts are produced using the former technique, which involves the use of specifically chosen strains of *lactic acid bacteria*. However, the production of other fermented foods, including kimchi and sauerkraut, depends on natural or wild bacteria [29].

The yogurt bacteria, *Streptococcus thermophilus* and *Lactobacillus delbrueckii* subsp., *Lactobacillus bulgaricus* (*L*. *bulgaricus*), have been found at the species level to help improve lactose digestion in people with lactose maldigestion, according to the European Food Safety Authority (EFSA Panel on Dietetic Products 2010), which established a health claim for yogurt [30].

Several epidemiological studies have supported the nutritional benefits of probiotics. For example, a reduced risk of metabolic syndrome was associated with yogurt-rich diets in one large cohort study of older adults [31]. Similarly, in another large cohort study, yogurt consumption was associated with less long-term weight gain [32]. The results of the Malmo diet and cancer cohort study showed that consumption of fermented dairy products (mainly yogurt and sour milk) was inversely associated with risk of cardiovascular disease [32]. Cheese consumption showed a similar effect, but only in women. Two large cross-sectional analyses of adults in Korea showed that high consumption (2–4 servings per day) of kimchi and other fermented foods and beverages was associated with reduced prevalence of atopic dermatitis [33]. Consumption of miso, natto, and fermented soy products was also inversely associated with reduced risk of high blood pressure [34]. Moreover, probiotic yogurt has been shown to benefit type 2 diabetes patients based on the findings of a study conducted on a group of individuals with the disease. The group who consumed probiotic yogurt containing *L. acidophilus La5* and *Bifidobacterium lactis Bb12* showed improvements in their fasting glucose levels and antioxidant capacity [35].

Recent clinical trials have shown that fermented milk can lower blood pressure in adults with hypertension [36], reduce infectious disease in children [37], and temporarily improve bone health markers in patients with osteoporosis [38]. Lastly, as is the case with cultured dairy products and other fermented meals, fermentation-related bacteria may raise the vitamin content of food [39].

### 3.2. Effect on Oral Health

A new focus has resulted in the emergence of prebiotics and probiotics for the delivery of relevant therapeutic health benefits. Further research has emerged for the use of either (or both) pre- and probiotic formats for non-gut applications, such as oral health products to protect against dental caries. The main focus of pre- and probiotics in oral health applications is to control cariogenic streptococci which colonizes the mouth [40].

*L. reuteri* is the probiotic species most often utilized in patients with chronic periodontitis. When taken as a pill once or twice a day, it has proven to be a helpful adjunct to scaling and root planing. The observed treatment outcomes included bleeding, probing pocket depth, gingival index, and plaque index reduction. Additionally, when healthy individuals were given a probiotic tablet containing *L. salivarius*, salivary buffering capacity was shown to increase. Based on these observations, the authors concluded that probiotic administration may result in enhanced resistance against caries [41,42]. Probiotic bacteriotherapy was investigated to determine the effect of chewing gums containing probiotic bacteria and xylitol on salivary *Streptococcus mutans* counts in 7–12-year-old children, and to assess whether there was a change in plaque and gingival scores after chewing these gums for 3 weeks. Xylitol chewing gum is widely used commercially. Several in vivo trials have proven the effectiveness of xylitol in the reduction of *Streptococcus mutans* counts in plaque and saliva. Xylitol, a five-carbon polyol, is metabolized via the intra-cellular phospho-enolpyruvate-phospho-transferase (PEP-PTS) pathway of *S. mutans*, whereas sucrose is metabolized via the glycolysis pathway. The PEP-PTS pathway converts xylitol to xylitol-5-phosphate, which competes with phosphofructokinase and then arrests glycolysis via intracellular accumulation of glucose 6-phosphate. Several investigations found that using xylitol or sorbitol alone significantly inhibited the production of *S. mutans* biofilms. On the other hand, biofilm quantification and imaging techniques show that sucrose supplementation dramatically reduced the inhibitory action of polyols, regardless of the presence of xylitol, sorbitol, or mixtures at 10% concentration. Therefore, it follows that if sucrose is regularly present in the oral cavity, xylitol-containing medical products, such chewing gum, will not be able to stop the development of dental plaque containing *S. mutans*. It was suggested that replacing fermentable sugars in the diet with other carbohydrates will help reduce dental caries following the seminal Turku sugar study [43].

In an in vitro study conducted recently, constant arginine administration improved the oral microbiota’s resistance to acidification and inhibited the growth of opportunistic infections [44,45]. It has been demonstrated that probiotics, or living microorganisms, can be used in biological plaque control methods as an alternative to chemical plaque treatment. This treatment’s mechanism involves antibacterial action as well as other processes, such as immune function modifications, nutritional competition, and modification of the oral environment. It does not involve bactericidal activity [46]. Probiotic bacteria have been shown to be more resilient in dairy products associated to sheep, where they can produce bioactive peptides. Probiotics need to attach to dental surfaces, create antimicrobial compounds against oral infections, alter the environment of the mouth, and lessen the inflammatory host response in order to be effective for oral disorders [47].

Certain *L. reuteri* strains have the potential to promote gingival wound healing by upregulating the neuropeptide hormone oxytocin. Probiotic *lactobacilli* supernatants have been demonstrated in vitro to increase human gingival fibroblasts’ synthesis of prostaglandin E2 (PGE2) in the presence of interleukin (IL)-1β, potentially hastening the healing of oral wounds [48]. A 4-week active intervention involving feeding patients with probiotic tablets containing *L. rhamnosus* and *Latilactobacillus curvatus* (L. curvatus) showed beneficial effects on gingival inflammation, gingival crevicular fluid flow, and the levels of supragingival plaque accumulation [49].

A commercial probiotic product (fermented milk) altered the taxonomic makeup of the saliva microbiome in several short-term ways. Compared to individuals who did not consume the probiotic drink, those who consumed it typically had more complex salivary microbial populations [50].

The three major areas of modern caries prevention strategies are plaque biofilm removal, food variables, and hosts. Probiotic therapy is an alternate caries prevention strategy that has just been tested on a group of preschoolers in Jeddah. Utilizing non-pathogenic endogenous or commensal bacteria to displace and replace pathogenic germs, probiotics have become a viable and natural alternative for treating infectious disorders [51].

### 3.3. Effect on Immune System

Probiotics and prebiotics influence the human immune system. They influence cellular metabolism, proliferation, and epithelial barrier functions, among their many other beneficial effects on health [52]. Early colonization by *Bacteroides* and *bifidobacterium* species may be critical for the development of immunological control, as the gut microbiome is a dynamic process. A mother’s diet, the use of antibiotics, the mode of birth, the surroundings, changes in the household and with the infants, and other factors can all have an impact on early life colonization [53]. The surface of immune cells contains toll-like receptors (TLR), whose activation changes in response to immune system responses, enabling them to discriminate between gut microbiota and pathogens. Through a variety of mechanisms, including altered mucus production, decreased bacterial adhesion, improved tight junctions, increased cell survival, and induction of defensins or IgA, probiotics and their effector molecules influence the gut barrier [54]. There are many different food products that include probiotics, including cereals, biscuits, breads, sauces, yogurts, and drinks. Depending on the ingredient and desired effect, the amount of probiotics in a typical meal might range from 2 to 20 g daily. Growing knowledge of the health-promoting qualities of probiotics and prebiotics, which improve gut health, lower the risk of disease, and can be utilized in therapy, has spurred interest in, and the creation of, functional foods that include both of these microorganisms [55].

### 3.4. Effect on Intestinal Health

For many years, probiotics have been used to improve intestinal health. By preserving the epithelial barrier, encouraging cell survival, boosting the synthesis of antibacterial agents and proteins that protect cells, boosting protective immune responses, and preventing the production of proinflammatory cytokines like IFN-γ, TNF-α, IL-4, and IL-13, probiotics can control the functions of the intestinal epithelium. Pathogenic organisms such as *Vibrio cholerae*, enteropathogenic *E. coli*, and *Clostridium perfringens*, along with their toxins, have the potential to disturb intestinal function. It has been noted that intestinal barrier functions can be seriously harmed by alcohol use and non-steroidal anti-inflammatory drug use [56].

Short-chain fatty acids (SCFAs), which are secreted by probiotics, have been proven to have a favorable effect on the function of the intestinal barrier. It has been discovered that SCFAs such as butyrate, propionate, and acetate demonstrate a protective effect against the ethanol-induced alteration of barrier function [57]. Ethanol raises metabolic stress and disrupts tight junctions (TJs) and epithelial cytoskeletons. By triggering AMP-activated protein kinase (AMPK) in Caco-2cells, SCFAs reduce metabolic stress and strengthen TJs. Bacterial-derived butyrate improved the function of the epithelial barrier and stabilized the hypoxia-inducible factor (HIF) by lowering O_2_ concentration [56,57]. A study using a mouse model of colitis induced by dextran sulfate sodium (DSS) showed that propionate strengthened the barrier function and lowered oxidative stress and inflammation. Different quantities of SCFAs produced by fermenting various dietary fibers have both strengthening and protecting effects on the function of the epithelium barrier. Acetate, which is produced by *B. longum* subsp. *infantis* 157F, aids in the host intestinal epithelial cells’ defense against the transfer of *Escherichia coli* O157:H7’s Shiga toxin from the gut lumen to the bloodstream [57].

Apart from their direct control over intestinal epithelial cells through probiotics or probiotic-derived functional factors, probiotics have also been observed to improve intestinal epithelial integrity by reestablishing the equilibrium of the gut microbiota profile. In a randomized controlled experiment, supplementing overweight persons with *Bifidobacterium animalis* subsp. *lactis 420* increased the levels of *Lactobacillus* and *Akkermansia* and promoted a lean metabolism. These findings highlight the significance of probiotics’ regulatory actions on gut microbiota in preserving intestinal epithelial homeostasis [57].

A well-known connection between probiotics and warfarin also exists. Antibiotics may alter the gut flora and cause reduced production of vitamin K. Indeed, intestinal bacteria are well known for producing vitamin K [58]. This translates into the common increase of warfarin effects in patients treated with broad spectrum antibiotics.

The gut microbiota of adults is rather stable and is altered by a variety of external variables, including stress, illnesses, radiation therapy, and drugs [59]. Probiotics also play an important role in maintaining the health of the upper airways, preventing dental caries, preventing tonsillitis, promoting urogenital health, and treating wounds and throat infections. Today, probiotics are available as dietary supplements and have been shown to be effective as “standard drug therapy” [60].

## 4. Role in Other Diseases

### 4.1. Obesity

Due to excessive food intake and absorption and reduced energy expenditure, obesity has become an epidemic clinical condition [61]. Numerous recent investigations have verified that human intestinal bacteria contribute significantly to the development of obesity, using efficient energy production and nutrition absorption. Additionally, it is still acknowledged that the duodenal microbiota in obese individuals is more diverse than in lean individuals. Obesity has been linked to the growth of some gut microbial taxa, including *Escherichia coli*, *Staphylococcus aureus*, and other generic bacterial species, such as *bifidobacterium* [62].

The early mechanism supposed to be accountable for such an upsurge in body fat was credited to the capability of microbiota to extract energy after food elements and control the energy balance of the host. Modification of dietary polysaccharides and fibers by firmicutes and Bacteroides in the gut results in the generation of short chain fatty acids, such as acetate, propionate, and butyrate. Propionate is a significant energy source for the host, making de novo glucose and lipids in the liver [63,64].

In a recent survey, 61 primary studies were assessed. There were differences in the gut microbial composition between overweight and non-obese subjects. Alteration was observed in energy homeostasis, with gut microbiota affecting dietary consumption and storage of lipids [65].

**Figure 2:**
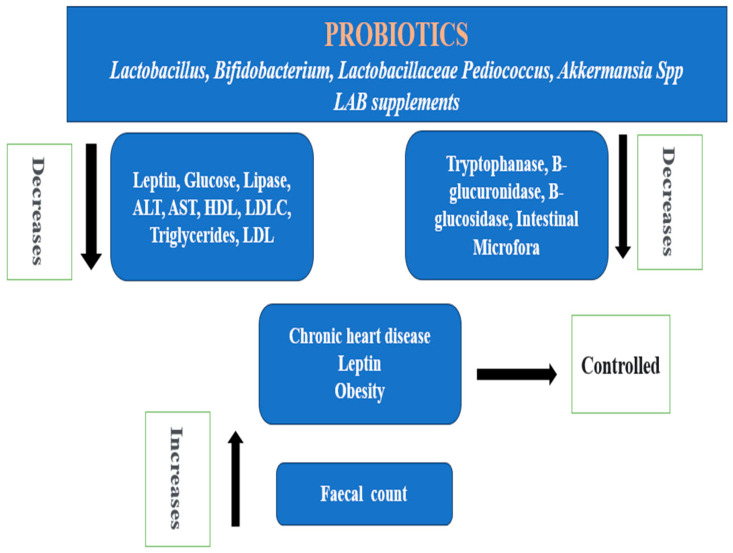
Probiotics against obesity [63,66,67,68].

Figure 2 explains the anti-obesity actions of *Lactobacillus*, *Bifidobacterium*, *Lactobacillaceae Pediococcus*, *Akkermansia* spp., and *LAB* probiotics supplements. These probiotics work by lowering the low-density lipoprotein (LDL) and total cholesterol level, assisting in the maintenance of a normal weight with low leptin levels, and reducing the incidence of chronic heart disease. While there is a reported increased fecal count, they also lower LDL-C, AST, ALT, HDL, glucose, lipase, and triglycerides. The intestinal microflora, as well as the production of β-glucuronidase, β-glucosidase, and tryptophanase, is likewise decreased by these probiotics [63,66,67,68].

### 4.2. Chronic Kidney Disease

Concern over probiotics and prebiotic addition grew as more cases of chronic kidney disease were reported [69]. Depending on the cause, the progression of chronic renal disease can lead to severe uremia at varying rates. Chronic kidney disease (CKD) may lead to hemodialysis, kidney transplantation, and peritoneal dialysis [70].

Investigations have shown that uremic toxins may promote the progression of kidney damage by breaking tubular cells [71]. In CKD, inflammation is a multifactorial phenotype [72]. Throughout the disease, probiotics have emerged as a possible remedy, curbing the gut microbiota to reduce uremic retention solutes and improve cardiovascular disease. The first goal of managing probiotics during CKD is uremic retention solute removal [73].

Hypercalciuria and hyperoxaluria are the main risk factors for the development of renal stones, which can seriously harm kidney function. Most of the oxalate formation occurs in the gut. *Lactobacilli* are administered as supplements in nephrolithiasis [74] and may help prevent stone formation and lower the risk of urolithiasis.

**Figure 3:**
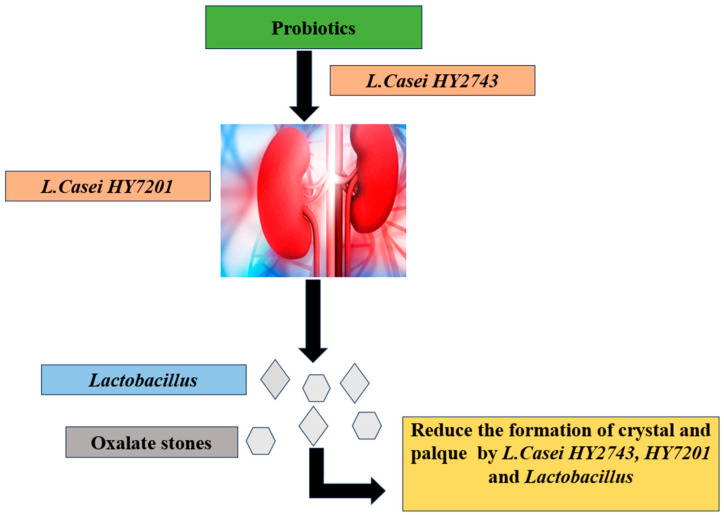
Prevention of kidney stones (oxalate) by probiotic (*Lactobacillus* spp.) [74].

According to a study by Cheol Kwak, *L casei HY2743* and *L. casei HY7201* can halt oxalate formation through various mechanisms [74]. These include the enzymatic degradation of oxalate by specific bacterial enzymes, such as oxalyl-CoA decarboxylase or formyl-CoA transferase, leading to the conversion of oxalate into harmless byproducts like formate and carbon dioxide. Additionally, these probiotic strains bind directly to oxalate molecules, preventing their intestinal absorption and promoting their fecal excretion, thus reducing systemic oxalate levels. Moreover, they could competitively inhibit oxalate absorption by occupying binding sites on intestinal epithelial cells [75]. Furthermore, by modulating the composition and function of the gut microbiota, *L. casei HY2743* and *L HY7201* might indirectly influence oxalate metabolism. As our understanding of the intricate interplay between gut microbiota and kidney stone formation continues to evolve, lactobacilli-based interventions could emerge as valuable tools in the comprehensive management of urolithiasis [74,75,76,77,78]. Figure 3 represents the mechanism of action of *Lactobacillus* spp. against oxalate stones.

### 4.3. Diabetes Mellitus

Diabetes mellitus is a major health problem worldwide [79] and is characterized by deficiency in insulin secretion and/or insulin action [80]. An autoimmune condition that results in the destruction of pancreatic beta cells causes type 1 diabetes, which is also referred to as insulin-dependent diabetes or juvenile onset diabetes [81].

In type 2 diabetes mellitus, the body does not secrete/utilize insulin correctly, and a condition of insulin resistance often exists [82].

Individuals with diabetes have altered gut microbiota. The human metagenome wide association study found a significant correlation between gut microorganisms, bacterial genes, and metabolic pathways in type 2 diabetes patients. When compared to non-diabetic patients, the amount of *Lactobacillus* spp. was dramatically different. Levels of fasting glucose and glycated hemoglobin (HbA1c) were favorably linked with the *Lactobacillus* species. *Clostridium* species had a negative relationship with fasting glucose, HbA1c, insulin, C-peptide, and plasma triglycerides, and a favorable relationship with adiponectin and high-density lipoprotein (HDL) cholesterol [83].

There is a 60% incidence of diabetic retinopathy in type 2 diabetes mellitus. Gram-positive bacteria and coagulase-negative staphylococci were discovered in diabetic subjects in higher proportions, mostly in retinopathy. The rate of *S. aureus* found in the subject’s eyes was larger than that found in healthy subjects and T1DM patients [84].

In T2D pathophysiology, the gut microbiota appears very important. Compositional and functional changes in gut microbiota are associated with the development of T2D. In studies on fecal samples, several mechanisms describe the influence of microbiota on T2DM development, including insulin resistance onset, short-chain fatty acids synthesis, metabolic endotoxemia, and alterations in the secretion of incretins. Many cytokines are involved in the progression of T2DM, and in the intestinal immune system there are fundamental signals affecting these phenomena [84].

Numerous pre-clinical studies have explained the impact of probiotics on glucose metabolism. These are mentioned in Table 1. A substantial body of research has indicated that the development of diabetes is associated with both oxidative damage and anti-oxidative capacity. Probiotic-induced inhibition of lipid peroxidation and increased synthesis of glutathione reduce oxidative damage in diabetic rats. Because probiotics have anti-diabetic qualities, they can help prevent insulin resistance. This ability also causes an increase in natural killer-T cells in the liver. Additionally, probiotics can reduce inflammation by modulating TNF-alpha expression, maintaining insulin stability, and inhibiting NF-kB tying activity. Probiotics may improve glucose metabolism also by increasing the bioavailability of some anti-diabetic drugs, such as gliclazide. They also aid in inhibiting intestinal glucose absorption and regulating autonomic nervous system activity [85].

Increases in the variety of microbial colonies support the integrity of the gastrointestinal lining, enhance glucose homeostasis, lessen inflammation, maintain insulin production, and improve nutrient absorption [86].

**Table 1:** Antidiabetic efficacy of probiotic interventions in pre-clinical studies [87,88].

Probiotics	Animal Model	Dose	Duration	Outcomes
*L. casei*	NOD mice	0.05% LC-containing diet	8 to 10 weeks	Improved blood glucose and host immune response
*L. rhamnosus* BSL *L. rhamnosus* R23	Streptozotocin induced diabetic rats	10^9^ CFU/mL	30 days	Reduced FBG and improved glucose tolerance via downregulation of glucose-6-phosphatase (G6pc)
Bulgarian *Lactobacillus* strains *Levilactobacillus brevis* (*L. brevis* 15) *L. plantarum* 13,	Fructose-induced diabetic rats	2.3–4.7 × 10^9^ cfu/mL L. brevis 15 and 0.7–1.5 × 10^9^ cfu/mL L. plantarum 13	8 weeks	Significantly lowered the blood glucose and HbA1c levels and free fatty acids and triglycerides.
Probiotic mixture *L. acidophilus* *Bifidobacterium. lactis* *L. rhamnosus*	Alloxan induced diabetic rats	75 mg/kg, equal quantities	3 days	Reduced blood glucose by improving gliclazide bioavailability in diabetic rats
*L*. *plantarum DSM 15313*	Female C57BL/6 J mice fed a high fat diet	25 × 10^8^ CFU/day	20 weeks	Lowered plasma glucose levels
*L. reuteri GMNL263*	STZ-induced diabetic rats	10^9^ CFU	28 days	Reduced glycated hemoglobin and blood glucose
*Bifidobacterium animalis subsp. lactis 420*	C57BL/6, ob/ob, CD14−/−, ob/obxCD14−/−, Myd88−/−, Nod1−/−or Nod2−/−mice fed a high fat diet	10^9^ CFU/day	6 weeks	Decreased TNF-α, IL-1β, PAI-1 and IL-6 Increased Insulin sensitivity
*Lactobacillus johnsonii N6.2 subsp. infanti ATCC*	Caco-2 cell/BB rats	10^10^ to 10^11^ CFU/L in cell culture and 10^8^ CFU/day in rats	-	Increased Paneth cells
*Lactobacillus Johnsonii* N6.2 *L. reuteri* TD1	BB rats, NOD mice, and C57BL/6 mice	1 × 10^8^ CFU/day	140 days	Positive TH17 phenotype modulation
*L. acidophilus*, *L. casei*, *L. lactis*	Male Wistar rats fed a high fructose diet	Dieet supplemented with 15% of dahi *ad libitum*	8 weeks	Decreased Blood glucose, HbA1c, glucose intolerance, plasma insulin, liver glycogen, plasma Tc, triacylglycerol, low-density lipoprotein cholesterol, blood free fatty acids

### 4.4. Autism

Gut problems are common in autistic children. Autism spectrum disorder (ASD) is a serious developmental and neurobehavioral disorder. ASD is associated with disturbances of the intestinal microbiota. Observational studies have shown alleviation of GI problems and/or improved behavioral traits with the provision of a gluten-free and casein-free diet. Tomova and colleagues evaluated the impact of 4 months of mixed probiotic administration on gut microbiota composition in ASD children and were able to show modulation of the Bacteroides ratio and an increase in bifidobacterial numbers [89]. Many gastrointestinal symptoms were linked to lower incidences when specific dietary components (such as a gluten or casein) were reduced in intake. Conversely, numerous research findings have demonstrated that probiotics, omega-3 fatty acids, vitamins, and mineral supplements, together with pharmacological and behavioral therapies, can make up for dietary deficits in autistic individuals [90]. Probiotics provided a potential therapeutic means for the treatment of *Clostridium* infections, but also resulted in decreased autistic features. These findings may open new perspectives in the therapeutic protocols associated with microbial health and potentially decrease the risk of developing autism. Oral probiotics prevented maternal immune activation-induced social deficits that are representative symptoms of ASD. Fructo-oligosaccharides (FOS) and maltodextrin were used for oral prebiotic administration [91]. A study of a combined probiotic–bovine colostrum product (BCP) supplementation was conducted in children with ASD. The gut microbiota’s alterations and supplement tolerability were the primary measures outcome. The study’s efficacy was assessed by tracking GI symptoms and any behavioral changes through metabolomic examination of plasma, urine, and feces. However, secondary outcome indicators were also examined, such as changes in the intestinal microbiota, cytokine expression in peripheral blood mononuclear cells (PBMCs), and host and microbial metabolism [92]. Probiotics produced vitamins and enzymes, altered the body’s acidic environment, and inhibited the growth of harmful bacteria. All things considered, their objective was to return the microbiota to its original, healthy form, which may have been impacted by environmental or nutritional factors like overuse of antibiotics. Gram-positive bacteria make up the majority of those often used probiotics for human consumption [93]. The fecal microbiota or urine metabolites were consistently improved by probiotic therapy, even with variations in dosage, species, strains, and duration [94].

### 4.5. Osteoporosis

Osteoporosis affects the skeletal system, causing poor bone mass density, skeletal system degradation, and increased susceptibility to fractures. The distal forearm, femur, and the spine are bones where fractures occur most commonly. Postmenopausal women are more likely to experience these problems [95]. Bone loss occurs in menopause as estrogens play a crucial role in the development and maintenance of bones [96]. Osteoporosis is a common condition, and many people are at high risk of having insufficient bone mass. Males and females can develop osteoporosis at any stage of life, but older females are more susceptible to it [97].

The regulation of bone mass is significantly influenced by gut bacteria. While changes in the immune system drive the influence of gut microbiota on bone mass, in a healthy state, it regulates osteoclastogenesis. Because bone-resorbing osteoclasts and bone-forming osteoblasts are continuously remodeling, osteoporosis develops when there is an imbalance in this process [98].

Osteoporosis and rheumatoid arthritis are two defiant bone and joint disorders that can be treated with probiotics as a medicinal agent [99]. Probiotics support the generation of antimicrobial peptides, maintenance of the luminal pH in the gut and mucus formation, as well as modulating the altering of the microbiota in the stomach driven by the host immune system [100]. Although probiotics have a variety of mechanisms for their effects on bones, their most subtle effects on bones are caused by their integration with vitamins. Vitamins D, C, K, and folate are linked to the metabolism of calcium and are essential for bone growth [101].

*L. reuteri* 6475 has a significant effect in reducing tumor necrosis factor in the host, which also limits bone absorption and maintains bone health. *L. reuteri* has a significant impact on bone density and osteoporosis [102]. After *B. longum* supplementation, there was an increase in bone resorption parameters, a decrease in serum C-terminal telopeptide (CTX) and osteoclasts, and a rise in osteocalcin (OC) serum levels and osteoblasts activity. *B. longum* administration modified the femoral bone structure [103].

Additionally, other *Lactobacillus* strains have demonstrated strong potential as a probiotic for bone health, such as *L. paracasei* and *L. brevis,* which prevent cortical bone loss in ovariectomized mice by reducing the production of two pro-inflammatory markers (TNF-α and IL-1β) and inhibiting osteoclastogenesis [104]. Moreover, oral administration of *L. rhamnosus GG* reduced gut permeability by promoting the expression of tight junction proteins and preventing the production of pro-inflammatory cytokines involved in bone resorption. It also increased the production of SCFAs, which benefits bone formation [105]. On the other hand, administration of *L. acidophilus*, *L. casei*, and *Bifidobacterium*, *Bacillus coagulans*, and *L. reuteri* significantly increased the level of serum calcium 1,25-(OH)_2_- vitamin D and protected bone loss in a rodent model [106]. Oral supplementation with *L. reuteri* also increased the serum level of 25-hydroxyvitamin D in humans [104].

The combination of *L. plantarum* and *B. longum* promotes serum levels of calcium, phosphorus, and osteocalcin, reduces the expression of TNF-α and downregulates the production of microbial LPS and enhancing the bone formation [107]. *B. vulgatus* supplementation minimizes dysbiosis, downregulates markers of inflammation such as TNF-α, and reduces microbial production of LPS, leading to improvement in the structure and the strength of the bones [108].

Bacterial antigens enter the intestine through the intestinal epithelial wall and trigger immune responses when postmenopausal osteoporosis bone loss results from low estrogen levels. Additionally, probiotics serve to maintain intestinal calcium absorption, control aberrant host immunological responses, increase the strength of the intestinal epithelium wall, restore intestinal microbial diversity to stop bone resorption, and aid in the latent creation of an estrogen-like chemical [109]. Increased mineral density and diversity in bones, and enhanced cortical and trabecular microstructure, are all facilitated by *L. acidophilus* probiotics. The host immune system is immunomodulated by a *lactobacillus* characteristic. *L. acidophilus* is responsible for the decreased expression of osteoclastogenic factors (IL-6, IL-17, TNF-α, and RANKL) and the increased expression of anti-osteoclastogenic factors. Additionally, *L. acidophilus* possesses therapeutic qualities that have an osteoprotective effect in postmenopausal osteoporosis through adjusting the balance of Treg-Th17 cells [110].

### 4.6. Allergy Prevention

As a hypersensitive immune system ailment, allergy is also known as type I hypersensitivity and is characterized as a disease following an immune system response to an antigen. Allergies now affect almost half of the population in North America and Europe, with an increasing incidence rate. These allergic responses are brought on by one or more common environmental chemicals, or antigens. The most frequent allergic reactions are hay fever, rhinitis, dermatitis, urticaria, angioedema, asthma, and hypersensitivity to foods, medications, and insects. Since the gut microbiome modulates the immune and inflammatory response, which in turn affects the development of sensitization and allergy, it is a promising therapeutic target for the management of allergic illnesses [111].

*L. Plantarum SY12* and L. plantarum SY11 probiotics reduce the synthesis of nitric oxide, tumor necrosis factor-α, T helper 2 related cytokines, cyclooxygenase-2, and inducible nitric oxide synthase, which results in significant anti-allergy effects [111,112]. According to another study, taking L. reuteri orally helped lessen allergic diarrhea and improve the cinonic microflora’s deteriorated profile. Additionally, it down-regulated the expression of GATA3, up-regulated the expression of TGF-b, IL-10, and Foxp3, reduced the production of T-helper 1 and 2 cytokines, and boosted the activation of mast cells and serum immunoglobulin E (IgE). These findings demonstrated that probiotics had anti-allergic properties through altering intestinal flora and boosting tolerogenic immune responses [113].

### 4.7. Irritable Bowel Syndrome (IBW)

Irritable bowel syndrome is linked to abnormalities in intestinal homeostasis. These abnormalities cause intestinal immune cells and epithelial cells to respond to the gut microbiota in an uncontrollable manner, which can lead to damage responses, such as fibrosis and ulcers. Prebiotics are important food ingredients that help support the growth of good bacteria, which is linked to changes in the microbiota of the intestines. For irritable bowel syndrome, probiotics and prebiotics have been shown to both be beneficial [114].

### 4.8. Wound Healing

Infected skin wounds and stomach ulcers have been shown to heal more quickly when probiotics are taken. In healthy skin, the skin microbiota functions as a protective barrier that can control the inflammatory response of the skin to small epidermal injuries by both increasing and reducing cytokine production [115]. Probiotics exhibit beneficial effects through a variety of mechanisms, including direct pathogen killing, enhanced epithelial barrier function, competitive displacement of pathogenic bacteria, and fibroblast activation [8]. Probiotics can also lower the bacterial load in an ulcer area, which is especially helpful for burn patients [116]. An injury to the skin disrupts the microbiota and increases the presence of bacteria that negatively impact wound closure. Wounds are stressful conditions that lead to changes in neuroendocrine function, infection, and poor wound healing [117].

Venous leg ulcers (VLU), decubitus ulcers (DU), and diabetic foot ulcers (DFU) are some examples of chronic wounds that are hard to heal and can be burdensome for the patient as well as the healthcare system. The formation of damaged wounds is largely due to the presence of polymicrobial biofilms in chronic wounds, which encourage the growth of harmful microorganisms and obstruct the healing process [118].

In addition to having a favorable impact on gut health, probiotics have been shown to improve skin-related issues like burns, scars, and infections. They also boost the skin’s natural immunity and aid in the regeneration of healthy skin [118].

### 4.9. Helicobacter Pylori Infection

*Helicobacter pylori* (*H. pylori*), is thought to infect over 4.4 billion people worldwide [119]. Compared to industrialized nations, developing nations are more likely to have *H. pylori* infections, with up to 80% of the population being infected. Probiotics can inhibit *H. pylori* in a competitive manner, serving as bacteriostatic agents and enhancing the gut microbiota. Probiotics such as *lactobacilli* and others, such as *Bifidobacterium*, *Bacillus licheniformis*, and *saccharomyces*, are currently in use and have demonstrated efficacy in treating gastrointestinal symptoms associated with *Helicobacter pylori* [120]. Several clinical trials, shown in Table 2, have reported decreasing rates in antibiotic-associated adverse events compared with probiotics, demonstrating an increase in *H. pylori* eradication. The activity of *H. pylori* urease can be suppressed by lactic acid. Moreover, probiotics’ production of reactive oxygen species damages the bacterial cell wall and its membranes [121].

Various components of the bacterial surface facilitate the adhesion of *H. pylori* to the gut epithelium. Probiotics have been shown in studies to enhance IgA synthesis, which fortifies the mucosal barrier’s defense against infections. Probiotics compete with *H. pylori* at microbial adhesion sites, increasing the immune response and counteracting its pathogenicity. The glycolipid-binding specificity of probiotics and *H. pylori* is presently being researched to determine the potential use of probiotics as anti-adhesion medications to treat *H. pylori*-induced stomach ulcers [119,122].

**Table 2:** Clinical trials that involve probiotic utilization to eradicate *H. pylori*.

Study	Probiotic	Outcomes	Reference
Randomized clinical trail	*Lactobacillus*	The success rate of *H. pylori* eradication is 100% in the probiotics group compared to 90% in the antibiotics group	[123]
Randomized clinical trail	*Bifidobacterium*, *Lactobacillus*	Reduced Fermicutes, Minimal effect on Proteobacteria, controlled antibiotic resistance in probiotic group, improve the *H. pylori* eradication success rate	[124]
open label single center study	*L. reuteri*	*H. pylori* eradication by accessing the urease activity before and after 4–6 weeks of therapy which reported a significant drop in *H. pylori* levels after probiotic administration,	[125]
prospective, multi-center, placebo-controlled study	Quadruple regimen of *L. acidophilus LA*-5, *Saccharomyces boulardii*, *L. plantarum*, *Bifidobacterium lactis* BB-12	*H. pylori*-eradication regimen increases the eradication rate and decreases side effects.	[126]
Randomized, double-blind, placebo-controlled	*L. reuteri* In bismuth-containing quadruple therapy	*H. pylori* eradication was found to be 85%. *Lim. reuteri* was able to reduce pain and abdominal distension	[120]

## 5. Side Effects Associated with Probiotics

Probiotics are generally considered safe for a healthy population, but specific subsets of the population with underlying medical conditions, such as systemic infections, deleterious metabolic activities, excessive immune stimulation, and gene transfer are highly sensitive to probiotics intake. The interactions between intestinal microbes and the host have a major influence on the overall health condition. Due to the fact that the adverse effects caused by probiotics are documented, and the suitable characteristics of relationships between probiotic structure and function would reduce the possibility of side effects, it is necessary to fully understand the mechanisms of activity of probiotic bacteria. However, the risks associated with the unrestricted dietary intake of probiotics have been listed in tabular form in Table 3 [127].

## 6. Conclusions

In the 20th century, probiotics were recognized as a significant factor in intestinal health and the presence of beneficial bacteria in the human gut became regarded as an important health asset. It was thought that these gut microorganisms were linked to longer lifespans. Research on the health-promoting qualities and mode of action of these gut microorganisms in the context of various common disorders is currently based on the correlation between probiotics and a longer life span.

Probiotics work to improve gut health through a variety of mechanisms. These include suppressing pro-inflammatory cytokines and stimulating anti-inflammatory cytokines to modulate immune responses; producing bioactive compounds like vitamins and short-chain fatty acids that support host health; and restoring the balance of gut microbes by competing with pathogens for resources and producing antimicrobial compounds. Moreover, probiotics also assist in metabolizing food ingredients to improve nutrition absorption and digestion; boosting mucosal immunity via secretory IgA antibody production; lowering gut inflammation via immune activity modulation and blocking pro-inflammatory signaling pathways; and promoting neurological health through the gut-brain axis, which produces neurotransmitters and neuroactive substances that affect mood and thought processes. Together, these effects support immune system performance, gastrointestinal health, and general wellbeing.

Researchers have found that *E. coli*, *Staphylococcus aureus*, *Bifidobacterium*, and Firmicutes can affect obesity and hence be exploited as an obesity treatment. One of the most common diseases in the world, type 2 diabetes, can also be affected with probiotics. Probiotics can be helpful in the prevention and management of diabetes due to their anti-diabetic and anti-inflammatory qualities. Children with autism have also shown benefit from probiotics, particularly in relation to the GIT dysfunctions associated with this condition. In oral, autoimmune, and allergy conditions, as well as respiratory syndromes and cancers, probiotics provide promising effects. Because gut microbes promote the improved absorption of several nutrients and minerals, it has also been demonstrated that they have a favorable impact on bone mineral density. Probiotic effects on various diseases and their mechanisms of action are summarized in Table 4. However, despite the multiple benefits of probiotics, some categories of individuals, such as immunocompromised patients, infants, elderly patients with comorbidities as well as critically ill patients, should avoid using probiotics.

Nevertheless, more research is needed to guarantee the safe use of specific bacterial strains because the available evidence is currently insufficient. As is well known, not all microorganisms are advantageous, and some can have negative effects. As a result, detailed investigations into specific strain usages, effects on health, and interactions are necessary. Physicians should not be urged to employ probiotic bacteria as a therapeutic avenue until there is compelling evidence to support their usage.

## Figures and Tables

**Table 3:** Observed adverse events associated with dietary intake of probiotics [127,128,129].

Probiotic Strain	Case Studies	Side Effects
*L. rhamnosus* GG	Pre-term infant with short gut syndrome	*Lactobacillus* bacteremia
	11-month-old infant with short gut syndrome	
	17-year-old boy with ulcerative colitis	
	24-year-old female cardiosurgical patient	Probiotic sepsis
	Critically ill children with antibiotic related diarrhea	Sepsis
	Children (up to 4 years)	Allergic rhinitis and more serious asthma
	Pregnant (4–6 weeks before expected delivery) and breastfeeding mothers (up to 2 years)	Wheezing bronchitis; atopic sensitization
*L. rhamnosus*	11-month-old female with trisomy 21 with respiratory viral infection	Probiotic associated pneumonia
	>65-year-old patient with hemorrhagic telangiectasia (HHT)	Endocarditis
	11-month-old female with trisomy 21 with respiratory viral infection	Probiotic associated pneumonia
*Lactobacillus* spp.	58-year-old immunocompetent with mechanical ventilation	*Lactobacillus* bacteremia and sepsis
		Unregulated ‘bile salt hydrolase (BSH)’ activity
*B. longum*	74-year-old man with polymetastatic prostatic adenocarcinoma	*Bifidobacterium* bacteremia
	Pre-term infants, Low birth-weight infants	
*B. breve*	2-year-old boy with Philadelphia chromosome-positive acute B-cell lymphoblastic leukemia	*Bifidobacterium* sepsis
*E. coli* NISSLE strain 1917	Pre-term infants	Severe sepsis
*Saccharomyces* *cerevisiae* var. *boulardii*	48-year-old diabetic with multiple co-morbidities	Multiple organ failure and septic shock in association with toxic megacolon and probiotic fungemia
	Immunocompromised 73-year-old patient on chemotherapy	Fungemia
	8-year-old boy with respiratory distress (Intensive care unit patient)	
	Critically ill patients	
	Premature neonate receiving nutrition enterally	Fungal septicemia
*L. plantarum*	30-year-old male with rheumatic valve disease	Endocarditis
*L. jensenii*	47-year-old immunocompetent patient	
*L. acidophilus*	48-year-old male with heart disease and dental manipulations	
*L. casei*	60-year-old with renal transplant patient	Intra-abdominal abscess
	53-year-old immunocompetent patient	Endocarditis
*L. paracasei*	65-year-old diabetic patient	Bacteremia and liver abscess
	77-year-old male patient with prostate cancer	Endocarditis
*L. acidophilus* LAVRI-A1	Infants (6 and 12 months)	Atopic sensitization
*L. acidophilus*, *L. casei*, *L. salivarius*, *L. lactis*, *B. bifidum*, and *B. lactis*	Critically ill patients (acute pancreatitis)	Increased local oxygen demand
*L. lactis*		Biogenic amine production
*Pediococcus* and *Leuconostoc* sp.		Vancomycin resistance → *Staphylococcus aureus*
*Lactobacillus* spp.		Broad spectrum antibiotic resistance (vancomycin, streptomycin, gentamicin, aztreonam, and ciprofloxacin) → *Staphylococcus* sp.

**Table 4:** Summary of probiotics’ impact and mode of action in preventing different diseases.

Probiotics	Type	Subject	Duration	Diseases	Effect	Mechanism of Action	Reference
*Lactobacillus*	*L. acidophilus*	Humans	6 weeks	Type 2 diabetes	Improved the glycemic control	Maintaining and decreasing insulin sensitivity. Decrease in inflammatory cytokines (TNF-α and resistin) and increase in the acetic acid.	[130]
	*L. reuteri*	20 older women	1 year	Low bone mineral denisty	Bone density improves and loss of femur and spinal bone is avoided. Prevents postmenopausal bone loss	By lowering osteo clastogenesis, T-cells produced signals that repress osteoclasts.	[131]
	*L. casei*	Neonates	12 months	Enteric Colonization	It is possible to prevent fungal diseases such intestinal colonization.	Changes in fungal ecology carry out the application of processes that may be used in the gut by LGG. A notable fungus exclusion and decrease in colonization potential are also associated with increased IgA mucosal responses.	[132]
	*L. rhamnosus* GR-1 *L. reuteri* RC-14)	pre-menopausal female	3 months	UTI	Urinary tract infection prevention, including vaginal flora infections and pregnancy problems brought on by UTIs, could be addressed	To show a decrease in urinary tract infections and to increase IgA, women are administered probiotics as vaginal suppositories.	[133]
	*L. gasseri*	Rats	12 weeks	Obesity	Consuming probiotics through supplements could help with weight gain issues and obesity.	Probiotics taken out of human breast milk have a noticeable impact on fat tissues. This may be accomplished by eliminating or drastically lowering the number of cells.	[134]
	*L* *. plantarum*	Mice	10 days	Spontaneous Colitis	It is typically preventable to avoid colitis and restoring the disturbed gut microbiota	Restore gut microbiota by increasing beneficial bacteria such as *Lactobacillus* and decreasing intestinal pathogenic bacteria like *Proteobacteria. L. plantarum*-12 administration could improve immunity *via* activating the janus kinase-signal transducer and the activator of the transcription (JAK-STAT) pathway and up-regulating adenosine deaminase (ADA) and interferon-induced protein with tetratricopeptide repeats 1 protein (IFIT1), and enforce the intestinal barrier function by up-regulating mucin 2 (MUC2) protein expression	[135]
	*L. rhamnosus R00 11/L.* *Helveticus ROO52*	Neonates	7 days	Rotavirus	It strengthen the immune system and rotavirus is prevented by building an effective immune system.	Because probiotics have anti-inflammatory qualities, they lower the danger of rotavirus when injected into IPEC-J2 cells.	[136]
*Bifidobacterium*	*B* *. lactis*	Mice	12 weeks	Obesity	Cut down on weight gain and fat mass	decreases mucosal bacterial adhesion in the ileum and caecum significantly	[137]
	*B. infantis*	Rat	10 days	Inflammatory bowel disease	help regulate atypical immune responses in the intestinal tissues	Decreases the invasion of lymphocytes and slows down the decrease of goblet cells	[138]
	*B* *. adolescentis*	Cells	NR	Melanogenesis	Because of its antioxidant and melanoma-inhibiting qualities, *B. adolescentis* is a unique skin-whitening product.	Reduced melanogenesis, such as the melanoma process in cells, would result from inhibition of tyrosinase activity.	[139]
	*B* *. infantis*	Mice	7 days	Inflammatory bowel disease	One potential treatment for IBD is to decrease intestinal permeability.	It is reported that there has been a decrease in neutrophil infiltration and inflammation in the colon.	[140]
	*Breve*	Mice	NR	Alzheimer’s disease	possess advantageous properties for peripheral tissues, the central nervous system, and the treatment of neurodegenerative diseases.	A particular probiotic reduces hippocampal expressions and inflammation, whereas non-viable microbe metabolites or their components partially alleviate cognitive deterioration.	[141]
	*B. longum*	murine mode	10 days	Influenza infection	Microbiota-based antiviral immune responses safeguard people who are most vulnerable to severe respiratory infection outcomes.	The cell wall of *B. longum* decreases type I IFN responses while increasing the proper type III interferons and surfactant protein D responses for antiviral defense.	[142]
Other species	*Escherichia coli*	Humans	12 weeks	Irritable bowel syndrome	The irritable bowel syndrome is lessened by EcN, which has positive consequences.	demonstrates its effectiveness especially in individuals with altered intestinal microbiota, such as those who have had gastro enterocolitis or have taken antibiotics.	[143]
	*Streptococcus thermophilus*	mice	9 days	Diarrhea	The fermentation-derived formula has the potential to lessen severe diarrhea.	Accelerated the recovery of the enlarged caecum and intestinal barrier injury from antibiotic associated diarrhea (AAD), and further decreased endotoxin (ET), D-lactate (D-LA) and diamine oxidase (DAO) content in serum. Moreover, pro-inflammatory cytokines (TNF-α) were reduced, while interferon-γ (IFN-γ) and anti-inflammatory cytokines (IL-10) increased after treating with *Streptococcus thermophiles* DMST-H2	[144]
	*Bacillus subtilis*	Rabbits	7 weeks	Immunodeficiency	Enhancement of immunity and defensive mechanisms	gives innate immunity and induces immunity in RK-13 cells, and it significantly increases the weight of the spleen and thymus.	[145]
	*Lactococcus lactis*	Mice	7 days	Ulcerative coliti	Epithelial tissue damage might be avoided and both acute and chronic colitis could be effectively treated.	Unlike pure TFF, which has been shown to repair and prevent acute colitis caused by DSS, *L. lactis*, which releases TFF, is engaged in the intragastric injection at the colonic mucosa.	[146]
	*Sporolactobacillus insulins*	Pigs	NR	Porcine edema	Porcine edema induction decreased weight gain and improved body metrics.	lowers the death rates of pigs brought on by STEC	[147]

NR = Not reported; EcN = Escherichia coli Nissle; STEC = Shiga toxin 2e-producing Escherichia coli; RK-13 cells = Rabbit Kidney Epithelial Cell line; TFF = Trefoil factors; DSS = Dextran sodium sulfate; LGG *= Lacticaseibacillus rhamnosus*; IgA = Immunoglobulin A; UTI = Urinary Tract Infection; IL = Interleukin; SPF = Specific pathogen-free; IPEC-J2 = non-transformed porcine jejunum epithelial cell line; IBD = Inflammatory bowel disease; AD = Alzheimer’s disease.

## Data Availability

No new data was created or analyzed in this study. Data sharing is not applicable to this article.

## References

[1] Ma T., Shen X., Shi X., Sakandar H.A., Quan K., Li Y., Jin H., Kwok L.-Y., Zhang H., Sun Z. (2023). Targeting gut microbiota and metabolism as the major probiotic mechanism—An evidence-based review. Trends Food Sci. Technol..

[2] Granato D., Branco G.F., Nazzaro F., Cruz A.G., Faria J.A. (2010). Functional foods and nondairy probiotic food development: Trends, concepts, and products. Compr. Rev. Food Sci. Food Saf..

[3] Ji J., Jin W., Liu S., Jiao Z., Li X. (2023). Probiotics, prebiotics, and postbiotics in health and disease. MedComm.

[4] Vera-Santander V.E., Hernández-Figueroa R.H., Jiménez-Munguía M.T., Mani-López E., López-Malo A. (2023). Health benefits of consuming foods with bacterial probiotics, postbiotics, and their metabolites: A review. Molecules.

[5] Dhopatkar N., Keeler J.L., Mutwalli H., Whelan K., Treasure J., Himmerich H. (2023). Gastrointestinal symptoms, gut microbiome, probiotics and prebiotics in anorexia nervosa: A review of mechanistic rationale and clinical evidence. Psychoneuroendocrinology.

[6] Feng P., Zhao S., Zhang Y., Li E. (2023). A review of probiotics in the treatment of autism spectrum disorders: Perspectives from the gut–brain axis. Front. Microbiol..

[7] Wang C., Bai J., Chen X., Song J., Zhang Y., Wang H., Suo H. (2023). Gut microbiome-based strategies for host health and disease. Crit. Rev. Food Sci. Nutr..

[8] Lukic J., Chen V., Strahinic I., Begovic J., Lev-Tov H., Davis S.C., Tomic-Canic M., Pastar I. (2017). Probiotics or pro-healers: The role of beneficial bacteria in tissue repair. Wound Repair Regen..

[9] Sakandar H.A., Zhang H. (2021). Trends in Probiotic(s)-Fermented milks and their in vivo functionality: A review. Trends Food Sci. Technol..

[10] Xavier-Santos D., Padilha M., Fabiano G.A., Vinderola G., Cruz A.G., Sivieri K., Antunes A.E.C. (2022). Evidences and perspectives of the use of probiotics, prebiotics, synbiotics, and postbiotics as adjuvants for prevention and treatment of COVID-19: A bibliometric analysis and systematic review. Trends Food Sci. Technol..

[11] Sanders M.E., Merenstein D.J., Reid G., Gibson G.R., Rastall R.A. (2019). Probiotics and prebiotics in intestinal health and disease: From biology to the clinic. Nat. Rev. Gastroenterol. Hepatol..

[12] Katkowska M., Garbacz K., Kusiak A. (2021). Probiotics: Should all patients take them?. Microorganisms.

[13] Rad A.H., Aghebati-Maleki L., Kafil H.S., Abbasi A. (2020). Molecular mechanisms of postbiotics in colorectal cancer prevention and treatment. Crit. Rev. Food Sci. Nutr..

[14] Yadav M.K., Kumari I., Singh B., Sharma K.K., Tiwari S.K. (2022). Probiotics, prebiotics and synbiotics: Safe options for next-generation therapeutics. Appl. Microbiol. Biotechnol..

[15] Palai S., Derecho C.M., Kesh S.S., Egbuna C., Onyeike P.C. (2020). Prebiotics, probiotics, synbiotics and its importance in the management of diseases. Functional Foods and Nutraceuticals: Bioactive Components, Formulations and Innovations.

[16] Patra F., Duary R., Ganguly S., Das A. (2017). Engineered probiotics and pharmabiotics: Application in therapeutics and prophylaxis. Indian J. Dairy Sci..

[17] Hojsak I., Kolaček S. (2024). Role of Probiotics in the Treatment and Prevention of Common Gastrointestinal Conditions in Children. Pediatr. Gastroenterol. Hepatol. Nutr..

[18] Fijan S. (2014). Microorganisms with claimed probiotic properties: An overview of recent literature. Int. J. Environ. Res. Public Health.

[19] Byakika S., Mukisa I.M., Byaruhanga Y.B., Muyanja C. (2019). A review of criteria and methods for evaluating the probiotic potential of microorganisms. Food Rev. Int..

[20] Williams N.T. (2010). Probiotics. Am. J. Health Syst. Pharm..

[21] Wolfe W., Xiang Z., Yu X., Li P., Chen H., Yao M., Fei Y., Huang Y., Yin Y., Xiao H. (2023). The challenge of applications of probiotics in gastrointestinal diseases. Adv. Gut Microbiome Res..

[22] Darbandi A., Asadi A., Ari M.M., Ohadi E., Talebi M., Zadeh M.H., Emamie A.D., Ghanavati R., Kakanj M. (2021). Bacteriocins: Properties and potential use as antimicrobials. J. Clin. Lab. Anal..

[23] Negash A.W., Tsehai B.A. (2020). Current applications of bacteriocin. Int. J. Microbiol..

[24] Sahadeva R.P., Leong S.F., Chua K.H., Tan C.H., Chan H.Y., Tong E.V., Wong S.Y., Chan H.K. (2011). Survival of commercial probiotic strains to pH and bile. Int. Food Res. J..

[25] Sharma N., Kang D.-K., Paik H.-D., Park Y.-S. (2022). Beyond probiotics: A narrative review on an era of revolution. Food Sci. Biotechnol..

[26] Dbeibia A., Mahdhi A., Jdidi S., AAltammar K., Zmanter T., Mzoughi R., Jabeur C. (2023). Probiotic potential of lactic acid bacteria isolated from colostrum of 3 different mammals. Food Biotechnol..

[27] Shewale R.N., Sawale P.D., Khedkar C.D., Singh A. (2014). Selection criteria for probiotics: A review. Int. J. Probiotics Prebiotics.

[28] Sanders M.E., Merenstein D., Merrifield C.A., Hutkins R. (2018). Probiotics for human use. Nutr. Bull..

[29] Marco M.L., Heeney D., Binda S., Cifelli C.J., Cotter P.D., Foligné B., Gänzle M., Kort R., Pasin G., Pihlanto A. (2017). Health benefits of fermented foods: Microbiota and beyond. Curr. Opin. Biotechnol..

[30] EFSA Panel on Dietetic Products, Nutrition and Allergies (NDA) (2010). Scientific Opinion on the substantiation of health claims related to live yoghurt cultures and improved lactose digestion (ID 1143, 2976) pursuant to Article 13(1) of Regulation (EC) No 1924/2006. EFSA J..

[31] Babio N., Becerra-Tomás N., Martínez-González M.Á., Corella D., Estruch R., Ros E., Sayón-Orea C., Fitó M., Serra-Majem L., Arós F. (2015). Consumption of yogurt, low-fat milk, and other low-fat dairy products is associated with lower risk of metabolic syndrome incidence in an elderly Mediterranean population. J. Nutr..

[32] Mozaffarian D., Hao T., Rimm E.B., Willett W.C., Hu F.B. (2011). Changes in diet and lifestyle and long-Term weight gain in women and men. N. Engl. J. Med..

[33] Park S., Bae J.-H. (2015). Fermented food intake is associated with a reduced likelihood of atopic dermatitis in an adult population (Korean National Health and Nutrition Examination Survey 2012–2013). Nutr. Res..

[34] Beltrán-Barrientos L.M., González-Córdova A.F., Hernández-Mendoza A., Torres-Inguanzo E.H., Astiazarán-García H., Esparza-Romero J., Vallejo-Cordoba B. (2018). Randomized double-blind controlled clinical trial of the blood pressure–lowering effect of fermented milk with *Lactococcus lactis*: A pilot study. J. Dairy Sci..

[35] Ejtahed H.S., Mohtadi-Nia J., Homayouni-Rad A., Niafar M., Asghari-Jafarabadi M., Mofid V. (2012). Probiotic yogurt improves antioxidant status in type 2 diabetic patients. Nutrition.

[36] Nozue M., Shimazu T., Sasazuki S., Charvat H., Mori N., Mutoh M., Sawada N., Iwasaki M., Yamaji T., Inoue M. (2017). Fermented soy product intake is inversely associated with the development of high blood pressure: The Japan public health center-based prospective study. J. Nutr..

[37] Nocerino R., Paparo L., Terrin G., Pezzella V., Amoroso A., Cosenza L., Cecere G., De Marco G., Micillo M., Albano F. (2017). Cow’s milk and rice fermented with *Lactobacillus paracasei* CBA L74 prevent infectious diseases in children: A randomized controlled trial. Clin. Nutr..

[38] Tu M.-Y., Chen H.-L., Tung Y.-T., Kao C.-C., Hu F.-C., Chen C.-M. (2015). Short-term effects of kefir-fermented milk consumption on bone mineral density and bone metabolism in a randomized clinical trial of osteoporotic patients. PLoS ONE.

[39] Chamlagain B., Sugito T.A., Deptula P., Edelmann M., Kariluoto S., Varmanen P., Piironen V. (2017). In situ production of active vitamin B12 in cereal matrices using *Propionibacterium freudenreichii*. Food Sci. Nutr..

[40] Meurman J.H., Stamatova I.V. (2018). Probiotics: Evidence of Oral Health Implications. Folia Med..

[41] Kaur K., Nekkanti S., Madiyal M., Choudhary P. (2018). Effect of chewing gums containing probiotics and xylitol on oral health in children: A randomized controlled trial. J. Int. Oral Health.

[42] Xu J., Chen C., Gan S., Liao Y., Fu R., Hou C., Yang S., Zheng Z., Chen W. (2023). The potential value of probiotics after dental implant placement. Microorganisms.

[43] Chan A., Ellepola K., Truong T., Balan P., Koo H., Seneviratne C.J. (2020). Inhibitory effects of xylitol and sorbitol on *Streptococcus mutans* and *Candida albicans* biofilms are repressed by the presence of sucrose. Arch. Oral Biol..

[44] Rosier B., Marsh P., Mira A. (2017). Resilience of the oral microbiota in health: Mechanisms that prevent dysbiosis. J. Dent. Res..

[45] Higuchi T., Suzuki N., Nakaya S., Omagari S., Yoneda M., Hanioka T., Hirofuji T. (2018). Effects of Lactobacillus salivarius WB21 combined with green tea catechins on dental caries, periodontitis, and oral malodor. Arch. Oral Biol..

[46] Nadelman P., Monteiro A., Balthazar C.F., Silva H.L., Cruz A.G., de Almeida Neves A., Fonseca-Gonçalves A., Maia L.C. (2019). Probiotic fermented sheep’s milk containing *Lactobacillus casei* 01: Effects on enamel mineral loss and *Streptococcus* counts in a dental biofilm model. J. Funct. Foods.

[47] Twetman S., Keller M.K., Lee L., Yucel-Lindberg T., Pedersen A.M.L. (2018). Effect of probiotic lozenges containing *Lactobacillus reuteri* on oral wound healing: A pilot study. Benef. Microbes.

[48] Keller M.K., Brandsborg E., Holmstrøm K., Twetman S. (2018). Effect of tablets containing probiotic candidate strains on gingival inflammation and composition of the salivary microbiome: A randomised controlled trial. Benef. Microbes.

[49] Dassi E., Ferretti P., Covello G., Bertorelli R., Denti M.A., De Sanctis V., Tett A., Segata N. (2018). The short-term impact of probiotic consumption on the oral cavity microbiome. Sci. Rep..

[50] AlAmoudi N.M., Almabadi E.S., El Ashiry E.A., El Derwi D.A. (2018). Effect of probiotic *Lactobacillus reuteri* on salivary cariogenic bacterial counts among groups of preschool children in jeddah, saudi arabia: A randomized clinical trial. J. Clin. Pediatr. Dent..

[51] Klaenhammer T.R., Kleerebezem M., Kopp M.V., Rescigno M. (2012). The impact of probiotics and prebiotics on the immune system. Nat. Rev. Immunol..

[52] Frei R., Akdis M., O’mahony L. (2015). Prebiotics, probiotics, synbiotics, and the immune system: Experimental data and clinical evidence. Curr. Opin. Gastroenterol..

[53] O’Flaherty S., Saulnier D., Pot B., Versalovic J. (2010). How can probiotics and prebiotics impact mucosal immunity?. Gut Microbes.

[54] Kerry R.G., Patra J.K., Gouda S., Park Y., Shin H.-S., Das G. (2018). Benefaction of probiotics for human health: A review. J. Food Drug Anal..

[55] Dahiya D., Nigam P.S. (2023). Nutraceuticals prepared with specific strains of probiotics for supplementing gut microbiota in hosts allergic to certain foods or their additives. Nutrients.

[56] Yan F., Polk D.B. (2020). Probiotics and probiotic-derived functional factors—Mechanistic insights into applications for intestinal homeostasis. Front. Immunol..

[57] Tripathy A., Dash J., Kancharla S., Kolli P., Mahajan D., Senapati S., Jena M.K. (2021). Probiotics: A Promising Candidate for Management of Colorectal Cancer. Cancers.

[58] Nath A., Haktanirlar G., Varga Á., Molnár M.A., Albert K., Galambos I., Koris A., Vatai G. (2018). Biological activities of *Lactose-derived* prebiotics and symbiotic with probiotics on gastrointestinal system. Medicina.

[59] Frazier T.H., DiBaise J.K., McClain C.J. (2011). Gut microbiota, intestinal permeability, obesity-induced inflammation, and liver injury. J. Parenter. Enter. Nutr..

[60] Ranjan A. (2022). The Use of Probiotics, Prebiotics, and Synbiotics as an Alternative to Antibiotics. Alternatives to Antibiotics: Recent Trends and Future Prospects.

[61] Dahiya D.K., Renuka, Puniya M., Shandilya U.K., Dhewa T., Kumar N., Kumar S., Puniya A.K., Shukla P. (2017). Gut microbiota modulation and its relationship with obesity using prebiotic fibers and probiotics: A review. Front. Microbiol..

[62] Ejtahed H.-S., Angoorani P., Soroush A.-R., Atlasi R., Hasani-Ranjbar S., Mortazavian A.M., Larijani B. (2018). Probiotics supplementation for the obesity management; A systematic review of animal studies and clinical trials. J. Funct. Foods.

[63] Vallianou N.G., Kounatidis D., Tsilingiris D., Panagopoulos F., Christodoulatos G.S., Evangelopoulos A., Karampela I., Dalamaga M. (2023). The role of next-generation probiotics in obesity and obesity-associated disorders: Current knowledge and future perspectives. Int. J. Mol. Sci..

[64] Mazloom K., Siddiqi I., Covasa M. (2019). Probiotics: How effective are they in the fight against obesity?. Nutrients.

[65] Sankararaman S., Noriega K., Velayuthan S., Sferra T., Martindale R. (2022). Gut microbiome and its impact on obesity and obesity-related disorders. Curr. Gastroenterol. Rep..

[66] Barathikannan K., Chelliah R., Rubab M., Daliri E.B.-M., Elahi F., Kim D.-H., Agastian P., Oh S.-Y., Oh D.H. (2019). Gut microbiome modulation based on probiotic application for anti-obesity: A review on efficacy and validation. Microorganisms.

[67] Ranjha M.M.A.N., Shafique B., Batool M., Kowalczewski P., Shehzad Q., Usman M., Manzoor M.F., Zahra S.M., Yaqub S., Aadil R.M. (2021). Nutritional and health potential of probiotics: A review. Appl. Sci..

[68] An H.M., Park S.Y., Lee D.K., Kim J.R., Cha M.K., Lee S.W., Lim H.T., Kim K.J., Ha N.J. (2011). Antiobesity and lipid-lowering effects of Bifidobacterium spp. in high fat diet-induced obese rats. Lipids Health Dis..

[69] Pei M., Wei L., Hu S., Yang B., Si J., Yang H., Zhai J. (2018). Probiotics, prebiotics and synbiotics for chronic kidney disease: Protocol for a systematic review and meta-analysis. BMJ Open.

[70] Ranganathan N., Friedman E.A., Tam P., Rao V., Ranganathan P., Dheer R. (2009). Probiotic dietary supplementation in patients with stage 3 and 4 chronic kidney disease: A 6-month pilot scale trial in Canada. Curr. Med. Res. Opin..

[71] Vitetta L., Gobe G. (2013). Uremia and chronic kidney disease: The role of the gut microflora and therapies with pro- and prebiotics. Mol. Nutr. Food Res..

[72] Mafra D., Fouque D. (2015). Gut microbiota and inflammation in chronic kidney disease patients. Clin. Kidney J..

[73] Koppe L., Mafra D., Fouque D. (2015). Probiotics and chronic kidney disease. Kidney Int..

[74] Kwak C., Jeong B.C., Ku J.H., Kim H.H., Lee J.J., Huh C.S., Baek Y.J., Lee S.E. (2006). Prevention of nephrolithiasis by Lactobacillus in stone-forming rats: A preliminary study. Urol. Res..

[75] Di Cerbo A., Pezzuto F., Palmieri L., Rottigni V., Iannitti T., Palmieri B. (2013). Clinical and experimental use of probiotic formulations for management of end-stage renal disease: An update. Int. Urol. Nephrol..

[76] Mogna L., Pane M., Nicola S., Raiteri E. (2014). Screening of different probiotic strains for their in vitro ability to metabolise oxalates: Any prospective use in humans?. J. Clin. Gastroenterol..

[77] Turroni S., Bendazzoli C., Dipalo S.C.F., Candela M., Vitali B., Gotti R., Brigidi P. (2010). Oxalate-degrading activity in *Bifidobacterium animalis* subsp. *lactis*: Impact of acidic conditions on the transcriptional levels of the oxalyl coenzyme A (CoA) decarboxylase and formyl-CoA transferase genes. Appl. Environ. Microbiol..

[78] Wigner P., Bijak M., Saluk-Bijak J. (2022). Probiotics in the Prevention of the Calcium Oxalate Urolithiasis. Cells.

[79] Guariguata L., Whiting D.R., Hambleton I., Beagley J., Linnenkamp U., Shaw J.E. (2014). Global estimates of diabetes prevalence for 2013 and projections for 2035. Diabetes Res. Clin. Pract..

[80] Kharroubi A.T., Darwish H.M. (2015). Diabetes mellitus: The epidemic of the century. World J. Diabetes.

[81] Miriam M.V. (2017). Psychosocio-immunogenetic factors in diabetic patients with vascular complications. Rev. Cuba. De Angiol. Y Cirugía Vasc..

[82] Yarnall A.J., Hayes L., Hawthorne G.C., Candlish C.A., Aspray T.J. (2011). Diabetes in care homes: Current care standards and residents’ experience. Diabet. Med..

[83] Blandino G., Inturri R., Lazzara F., Di Rosa M., Malaguarnera L. (2016). Impact of gut microbiota on diabetes mellitus. Diabetes Metab..

[84] Zhang Y., Zhang H. (2013). Microbiota associated with type 2 diabetes and its related complications. Food Sci. Hum. Wellness.

[85] Valdes A.M., Walter J., Segal E., Spector T.D. (2018). Role of the gut microbiota in nutrition and health. BMJ.

[86] Taylor B.L., Woodfall G.E., Sheedy K.E., O’riley M.L., Rainbow K.A., Bramwell E.L., Kellow N.J. (2017). Effect of probiotics on metabolic outcomes in pregnant women with gestational diabetes: A systematic review and meta-analysis of randomized controlled trials. Nutrients.

[87] Panwar H., Rashmi H.M., Batish V.K., Grover S. (2013). Probiotics as potential biotherapeutics in the management of type 2 diabetes—Prospects and perspectives. Diabetes/Metab. Res. Rev..

[88] Gomes A.C., Bueno A.A., de Souza R.G.M., Mota J.F. (2014). Gut microbiota, probiotics and diabetes. Nutr. J..

[89] Grimaldi R., Gibson G.R., Vulevic J., Giallourou N., Castro-Mejía J.L., Hansen L.H., Leigh Gibson E., Nielsen D.S., Costabile A. (2018). A prebiotic intervention study in children with autism spectrum disorders (ASDs). Microbiome.

[90] Cristiano C., Lama A., Lembo F., Mollica M.P., Calignano A., Mattace Raso G. (2018). Interplay Between Peripheral and Central Inflammation in Autism Spectrum Disorders: Possible Nutritional and Therapeutic Strategies. Front. Physiol..

[91] Wang X., Yang J., Zhang H., Yu J., Yao Z. (2019). Oral probiotic administration during pregnancy prevents autism-related behaviors in offspring induced by maternal immune activation via anti-inflammation in mice. Autism Res..

[92] Sanctuary M.R., Kain J.N., Chen S.Y., Kalanetra K., Lemay D.G., Rose D.R., Yang H.T., Tancredi D.J., German J.B., Slupsky C.M. (2019). Pilot study of probiotic/colostrum supplementation on gut function in children with autism and gastrointestinal symptoms. PLoS ONE.

[93] El-Ansary A., Al Dera H., Aldahash R. (2018). Effect of diet on gut microbiota as an etiological factor in autism spectrum disorder. Diet, Microbiome and Health.

[94] Hung L.Y., Margolis K.G. (2023). Autism spectrum disorders and the gastrointestinal tract: Insights into mechanisms and clinical relevance. Nat. Rev. Gastroenterol. Hepatol..

[95] Eastell R. (2016). Identification and management of osteoporosis in older adults. Medicine.

[96] Ahire J.J., Kumar V., Rohilla A. (2023). Understanding osteoporosis: Human bone density, genetic mechanisms, gut microbiota, and future prospects. Probiotics Antimicrob. Proteins.

[97] Ohlsson C., Sjögren K. (2015). Effects of the gut microbiota on bone mass. Trends Endocrinol. Metab..

[98] Yousf H., Tomar G.B., Srivastava R.K. (2015). Probiotics and bone health: It takes guts to improve bone density. Int. J. Immunother. Cancer Res..

[99] Collins F.L., Rios-Arce N.D., Schepper J.D., Parameswaran N., McCabe L.R. (2017). The potential of probiotics as a therapy for osteoporosis. Microbiol. Spectr..

[100] Parvaneh K., Jamaluddin R., Karimi G., Erfani R. (2014). Effect of probiotics supplementation on bone mineral content and bone mass density. Sci. World J..

[101] McCabe L.R., Irwin R., Schaefer L., Britton R.A. (2013). Probiotic use decreases intestinal inflammation and increases bone density in healthy male but not female mice. J. Cell. Physiol..

[102] Parvaneh K., Ebrahimi M., Sabran M.R., Karimi G., Hwei A.N.M., Abdul-Majeed S., Ahmad Z., Ibrahim Z., Jamaluddin R. (2015). Probiotics (*Bifidobacterium longum*) increase bone mass density and upregulate *Sparc* and *Bmp-2* genes in rats with bone loss resulting from ovariectomy. BioMed Res. Int..

[103] Xu X., Jia X., Mo L., Liu C., Zheng L., Yuan Q., Zhou X. (2017). Intestinal microbiota: A potential target for the treatment of postmenopausal osteoporosis. Bone Res..

[104] Duffuler P., Bhullar K.S., Wu J. (2024). Targeting gut microbiota in osteoporosis: Impact of the microbial based functional food ingredients. Food Sci. Hum. Wellness.

[105] Malik T.A., Chassaing B., Tyagi A.M., Vaccaro C., Luo T., Adams J., Darby T.M., Weitzmann M.N., Mulle J.G., Gewirtz A.T. (2016). Sex steroid deficiency–associated bone loss is microbiota dependent and prevented by probiotics. J. Clin. Investig..

[106] Tyagi A.M., Yu M., Darby T.M., Vaccaro C., Li J.-Y., Owens J.A., Hsu E., Adams J., Weitzmann M.N., Jones R.M. (2018). The microbial metabolite BUTYRATE stimulates bone formation via T regulatory cell-mediated regulation of WNT10B expression. Immunity.

[107] Kim D.-E., Kim J.-K., Han S.-K., Jang S.-E., Han M.J., Kim D.-H. (2019). *Lactobacillus plantarum* NK3 and *Bifidobacterium longum* NK49 alleviate bacterial vaginosis and osteoporosis in mice by suppressing NF-*κ*B-linked TNF-*α* expression. J. Med. Food.

[108] Yuan S., Shen J. (2021). Bacteroides vulgatus diminishes colonic microbiota dysbiosis ameliorating lumbar bone loss in ovariectomized mice. Bone.

[109] Dar H.Y., Shukla P., Mishra P.K., Anupam R., Mondal R.K., Tomar G.B., Sharma V., Srivastava R.K. (2018). *Lactobacillus acidophilus* inhibits bone loss and increases bone heterogeneity in osteoporotic mice via modulating Treg-Th17 cell balance. Bone Rep..

[110] Lamsal B.P. (2012). Production, health aspects and potential food uses of dairy prebiotic galactooligosaccharides. J. Sci. Food Agric..

[111] Latif A., Shehzad A., Niazi S., Zahid A., Ashraf W., Iqbal M.W., Rehman A., Riaz T., Aadil R.M., Khan I.M. (2023). Probiotics: Mechanism of action, health benefits and their application in food industries. Front. Microbiol..

[112] Lee N.-K., Kim S.-Y., Han K.J., Eom S.J., Paik H.-D. (2014). Probiotic potential of *Lactobacillus* strains with anti-allergic effects from kimchi for yogurt starters. LWT.

[113] Huang C.-H., Lin Y.-C., Jan T.-R. (2017). *Lactobacillus reuteri* induces intestinal immune tolerance against food allergy in mice. J. Funct. Foods.

[114] Horvat I.B., Gobin I., Kresović A., Hauser G. (2021). How can probiotic improve irritable bowel syndrome symptoms?. World J. Gastrointest. Surg..

[115] Nole K.L.B., Yim E., Keri J.E. (2014). Probiotics and prebiotics in dermatology. J. Am. Acad. Dermatol..

[116] Tsiouris C.G., Tsiouri M.G. (2017). Human microflora, probiotics and wound healing. Wound Med..

[117] Johnson T.R., Gómez B.I., McIntyre M.K., Dubick M.A., Christy R.J., Nicholson S.E., Burmeister D.M. (2018). The cutaneous microbiome and wounds: New molecular targets to promote wound healing. Int. J. Mol. Sci..

[118] Habeebuddin M., Karnati R.K., Shiroorkar P.N., Nagaraja S., Asdaq S.M.B., Anwer K., Fattepur S. (2022). Topical probiotics: More than a skin deep. Pharmaceutics.

[119] de Brito B.B., da Silva F.A.F., Soares A.S., Pereira V.A., Santos M.L.C., Sampaio M.M., Neves P.H.M., de Melo F.F. (2019). Pathogenesis and clinical management of *Helicobacter pylori* gastric infection. World J. Gastroenterol..

[120] Mestre A., Narayanan R.S., Rivas D., John J., Abdulqader M.A., Khanna T., Chakinala R.C., Gupta S. (2022). Role of Probiotics in the Management of *Helicobacter pylori*. Cureus.

[121] Lionetti E., Indrio F., Pavone L., Borrelli G., Cavallo L., Francavilla R. (2010). Role of probiotics in pediatric patients with *Helicobacter pylori* infection: A comprehensive review of the literature. Helicobacter.

[122] Cekin A.H., Sahinturk Y., Harmandar F.A., Uyar S., Yolcular B.O., Cekin Y. (2017). Use of probiotics as an adjuvant to sequential H. pylori eradication therapy: Impact on eradication rates, treatment resistance, treatment-related side effects, and patient compliance. Turk. J. Gastroenterol..

[123] Oh B., Kim B., Kim J.W., Kim J.S., Koh S., Kim B.G., Lee K.L., Chun J. (2015). The Effect of probiotics on gut microbiota during the *Helicobacter pylori* eradication: Randomized controlled Trial. Helicobacter.

[124] Yuan Z., Xiao S., Li S., Suo B., Wang Y., Meng L., Liu Z., Yin Z., Xue Y., Zhou L. (2021). The impact of *Helicobacter pylori* infection, eradication therapy, and probiotics intervention on gastric microbiota in young adults. Helicobacter.

[125] Dore M.P., Cuccu M., Pes G.M., Manca A., Graham D.Y. (2013). *Lactobacillus reuteri* in the treatment of *Helicobacter pylori* infection. Intern. Emerg. Med..

[126] Viazis N., Argyriou K., Kotzampassi K., Christodoulou D.K., Apostolopoulos P., Georgopoulos S.D., Liatsos C., Giouleme O., Koustenis K., Veretanos C. (2022). A four-probiotics regimen combined with a standard *Helicobacter pylori*-eradication treatment reduces side effects and increases eradication rates. Nutrients.

[127] Zawistowska-Rojek A., Tyski S. (2018). Are probiotic really safe for humans?. Pol. J. Microbiol..

[128] Kothari D., Patel S., Kim S.-K. (2018). Probiotic supplements might not be universally-effective and safe: A review. Biomed. Pharmacother..

[129] Dore M.P., Bibbò S., Fresi G., Bassotti G., Pes G.M. (2019). side effects associated with probiotic use in adult patients with inflammatory bowel disease: A systematic review and meta-analysis of randomized controlled trials. Nutrients.

[130] Tonucci L.B., dos Santos K.M.O., de Oliveira L.L., Ribeiro S.M.R., Martino H.S.D. (2017). Clinical application of probiotics in type 2 diabetes mellitus: A randomized, double-blind, placebo-controlled study. Clin. Nutr..

[131] Li P., Ji B., Luo H., Sundh D., Lorentzon M., Nielsen J. (2022). One-year supplementation with *Lactobacillus reuteri* ATCC PTA 6475 counteracts a degradation of gut microbiota in older women with low bone mineral density. npj Biofilms Microbiomes.

[132] Manzoni P., Mostert M., Leonessa M.L., Priolo C., Farina D., Monetti C., Latino M.A., Gomirato G. (2006). Oral supplementation with *Lactobacillus casei* subspecies *rhamnosus* prevents enteric colonization by candida species in preterm neonates: A randomized study. Clin. Infect. Dis..

[133] Wolff B.J., Price T.K., Joyce C.J., Wolfe A.J., Mueller E.R. (2019). Oral probiotics and the female urinary microbiome: A double-blinded randomized placebo-controlled trial. Int. Urol. Nephrol..

[134] Kang J.-H., Yun S.-I., Park H.-O. (2010). Effects of Lactobacillus gasseri BNR17 on body weight and adipose tissue mass in diet-induced overweight rats. J. Microbiol..

[135] Sun M., Liu Y., Song Y., Gao Y., Zhao F., Luo Y., Qian F., Mu G., Tuo Y. (2020). The ameliorative effect of *Lactobacillus plantarum*-12 on DSS-induced murine colitis. Food Funct..

[136] Liu F., Li G., Wen K., Bui T., Cao D., Zhang Y., Yuan L. (2010). Porcine small intestinal epithelial cell line (IPEC-J2) of rotavirus infection as a new model for the study of innate immune responses to rotaviruses and probiotics. Viral Immunol..

[137] Stenman L., Waget A., Garret C., Klopp P., Burcelin R., Lahtinen S. (2014). Potential probiotic *Bifidobacterium animalis* ssp. *lactis* 420 prevents weight gain and glucose intolerance in diet-induced obese mice. Benef. Microbes.

[138] Javed N.H., Alsahly M.B., Khubchandani J. (2016). Oral Feeding of Probiotic *Bifidobacterium infantis*: Colonic Morphological Changes in Rat Model of TNBS-Induced Colitis. Scientifica.

[139] Huang H.-C., Chang T.-M. (2012). Antioxidative properties and inhibitory effect of *Bifidobacterium adolescentis* on melanogenesis. World J. Microbiol. Biotechnol..

[140] Elian S., Souza E., Vieira A., Teixeira M., Arantes R., Nicoli J., Martins F. (2015). Bifidobacterium longum subsp. *infantis* BB-02 attenuates acute murine experimental model of inflammatory bowel disease. Benef. Microbes.

[141] Kobayashi Y., Sugahara H., Shimada K., Mitsuyama E., Kuhara T., Yasuoka A., Kondo T., Abe K., Xiao J.-Z. (2017). Therapeutic potential of *Bifidobacterium breve strain* A1 for preventing cognitive impairment in Alzheimer’s disease. Sci. Rep..

[142] Matsumoto T., Ishikawa H., Tateda K., Yaeshima T., Ishibashi N., Yamaguchi K. (2008). Oral administration of Bifidobacterium longum prevents gut-derived Pseudomonas aeruginosa sepsis in mice. J. Appl. Microbiol..

[143] Kruis W., Chrubasik S., Boehm S., Stange C., Schulze J. (2011). A double-blind placebo-controlled trial to study therapeutic effects of probiotic *Escherichia coli* Nissle 1917 in subgroups of patients with irritable bowel syndrome. Int. J. Color. Dis..

[144] Hu J.-S., Huang Y.-Y., Kuang J.-H., Yu J.-J., Zhou Q.-Y., Liu D.-M. (2020). *Streptococcus thermophiles* DMST-H2 promotes recovery in mice with antibiotic-associated diarrhea. Microorganisms.

[145] Guo M., Wu F., Hao G., Qi Q., Li R., Li N., Wei L., Chai T. (2017). *Bacillus subtilis* improves immunity and disease resistance in rabbits. Front. Immunol..

[146] Komaki S., Haque A., Miyazaki H., Matsumoto T., Nakamura S. (2020). Unexpected effect of probiotics by *Lactococcus lactis* subsp. lactis against colitis induced by dextran sulfate sodium in mice. J. Infect. Chemother..

[147] Tsukahara T., Inoue R., Nakanishi N., Nakayama K., Matsubara N., Ushida K. (2007). Evaluation of the low dose level of a heat-killed and dried cell preparation of *Enterococcus faecalis* to prevent porcine edema disease using experimental infection model with enterotoxcemic *Escherichia coli* in weaning pigs. J. Vet. Med. Sci..

